# Ameliorative effects of curcumin and resveratrol following abamectin-induced liver injury in rats

**DOI:** 10.1038/s41598-026-54518-9

**Published:** 2026-05-26

**Authors:** Neziha Hacihasanoğlu Çakmak, Bircan Kolbaşi, Talat Yasin Acar, İlknur Keskin, Türkan Yiğitbaşi

**Affiliations:** 1 Istanbul Medipol University, Vocational School of Health Services, Medical Laboratory Techniques, Istanbul, Türkiye; 2 School of Medicine, Department of Histology and Embryology, Istanbul Medipol University, Istanbul, Türkiye; 3 Research Institute for Health Science and Technologies, Istanbul, Türkiye; 4 School of Medicine, Department of Basic Medical Sciences, Department of Medical Biochemistry, Istanbul Medipol University, Istanbul, Türkiye

**Keywords:** Antioxidant effects, Oxidative stress, Liver, Curcumin, Resveratrol, Abamectin, Histopathology, Biochemistry, Drug discovery, Medical research, Zoology

## Abstract

Abamectin (ABA), a macrocyclic lactone of the avermectin family, is widely used in agriculture and animal husbandry for its insecticidal and anthelmintic properties. The present study investigated the therapeutic and antioxidant potential of curcumin (CUR) and resveratrol (RES) following abamectin-induced hepatotoxicity in Wistar albino rats. Fifty-eight male rats were randomly assigned to six groups: control (olive oil), CUR, RES, ABA, ABA + CUR, and ABA + RES. Treatments were administered orally for seven days, and liver tissues were collected on day eight for biochemical and histopathological evaluation. ABA exposure significantly decreased hepatic glutathione (GSH), total antioxidant capacity (TAC), and glutathione S-transferase (GST) activity, while markedly increasing lipid peroxidation (LPO), total oxidant status (TOS), and oxidative stress index (OSI) (p < 0.001). CUR and RES supplementation effectively mitigated these alterations, restoring antioxidant parameters and reducing oxidative stress. Histopathological analysis confirmed severe hepatic injury and elevated TNF-α expression in the ABA group, whereas CUR and RES treatments improved liver architecture and reduced injury scores. These results indicate that CUR and RES effectively attenuate ABA-induced oxidative liver damage and may represent promising adjunctive candidates for managing pesticide-related hepatotoxicity.

**Supplementary Information:**

The online version contains supplementary material available at 10.1038/s41598-026-54518-9.

## Introduction

Pesticides are chemical substances of natural or synthetic origin widely used in agricultural processes to improve crop yields and control pest populations; however, their extensive use raises serious concerns regarding adverse effects on human and animal health^1^. Among these compounds, abamectin (ABA), a macrocyclic lactone disaccharide produced through fermentation by Streptomyces avermitilis, is extensively utilized for its potent insecticidal and anthelmintic activities. It is broadly applied to crops such as cotton, citrus, corn, potatoes, and beans, and is also used in veterinary medicine to control parasitic infections in livestock^2^^,^^3^.

Despite its agricultural utility, ABA raises significant concerns regarding hepatotoxic effects in mammals due to its bioaccumulative nature and capacity to generate oxidative stress. Oxidative stress arises when the generation of reactive oxygen species (ROS) exceeds the capacity of the endogenous antioxidant defense system, leading to damage of cellular structures including membranes, organelles, proteins, and DNA, and ultimately promoting apoptosis and inflammatory responses^4^. Studies have specifically implicated oxidative stress as a major contributor to avermectin-induced cytotoxicity^5^, underscoring the need for effective antioxidant intervention to mitigate pesticide-induced tissue injury.

At the mechanistic level, abamectin exerts its biological effects primarily through modulation of γ-aminobutyric acid (GABA)-gated chloride channels. As a macrocyclic lactone of the avermectin family, ABA enhances inhibitory neurotransmission by increasing chloride ion influx via GABA receptors, leading to neuronal hyperpolarization and paralysis in target organisms^6^. Although this mechanism underlies its insecticidal and anthelmintic efficacy, excessive exposure may lead to neurotoxicity in mammals, particularly at high doses where macrocyclic lactones can cross the blood–brain barrier. Beyond neurotoxicity, experimental studies have demonstrated that ABA exposure is associated with increased reactive oxygen species production, lipid peroxidation, mitochondrial dysfunction, and disruption of cellular antioxidant defenses, linking its primary neuroactive properties with secondary systemic effects such as hepatic oxidative injury^5^^,^^7^.

Curcumin (CUR), a polyphenolic compound derived from the rhizome of *Curcuma longa* Linn (family Zingiberaceae), exhibits powerful antioxidant, anti-inflammatory, antibacterial, and anticancer activities^8^. CUR acts by scavenging a wide array of ROS — including superoxide anions $\left(O_2\cdot^{-}\right)$, peroxynitrite $\left(\rm{ONOO}^{-}\right)$, nitric oxide $\left(\rm{NO}\cdot\right)$, hydroperoxyl radicals $\left(\rm{HO}_2\cdot\right)$, and hydroxyl radicals $\left(\cdot\rm{OH}\right)$ — primarily through the action of its phenolic groups^9^. A growing body of evidence has documented CUR’s broad pharmacological profile, encompassing antioxidant^10^ anti-inflammatory^11^ antifibrotic^12^ anticancer^13,14^ anti-apoptotic^15^ and immunotherapeutic^16^ properties, collectively highlighting its promise as a therapeutic agent across diverse pathological conditions.

Similarly, resveratrol (RES), a natural polyphenolic phytoalexin present in grapes, red wine, and various plant-derived food products, exerts strong antioxidant, anti-carcinogenic, anti-inflammatory, and anti-aging effects. It modulates multiple signaling pathways and demonstrates antiviral, antimicrobial, neuroprotective, and cardioprotective functions^17^. The protective effects of both CUR and RES have been demonstrated against various toxic agents, including radiation, cadmium, and cisplatin^18^^,^^19^^,^^20^.

However, their comparative and combined efficacy against ABA-induced hepatotoxicity remains poorly defined, making this area of investigation both timely and scientifically relevant given the continued widespread use of ABA in agriculture. Therefore, the present study aimed to comparatively investigate the therapeutic effects of CUR and RES following ABA-induced liver damage in a rat model, employing a multi-layered approach that integrates antioxidant enzyme activities — including reduced glutathione (GSH), superoxide dismutase (SOD), catalase (CAT), glutathione reductase (GR), glutathione peroxidase (GPx), and glutathione-S-transferase (GST) — alongside total oxidant status (TOS), total antioxidant capacity (TAC), and TNF-α immunohistochemical staining.

## Materials and methods

### Chemicals and reagents

ABA, CUR, and RES were obtained from Cayman Chemical Company (Ann Arbor, MI, USA): ABA (CAS: 71751‑41‑2; ≥ 95%), CUR (CAS: 458‑37‑7; ≥ 90%), and RES (CAS: 501‑36‑0; ≥ 98%). TOS and TAC kits were obtained from FEIYUE Biotechnology (Wuhan, China). All other chemicals used were of analytical or HPLC grade. No further purification was performed.

### The experimental protocols

Fifty-eight Wistar albino rats, 8 weeks old and weighing 200 g on average, were used in the study. The animals were maintained in accordance with the ethical and welfare standards set by the Istanbul Medipol Institutional Animal Care and Use Committee (IMU-HADYEK) with the approved reference number (E-38828770–302.14.06–4079). Fifty-eight male Wistar albino rats were randomly allocated to six experimental groups using a computer-generated randomization method. The animals were housed in standard cages under optimal temperature (20 ± 2 °C) and light/dark (12 h light/12 h dark) conditions.

First group: control group rats were given 2 ml of olive oil daily (n = 10). In the second group, rats were given 200 mg/kg CUR daily for 7 days (n = 10)^21^. In the third group, rats were given 20 mg/kg RES daily for 7 days (n = 10)^22^. The fourth group included rats that received 2 mg/kg ABA daily for 7 days (n = 10)^23^. The fifth group included rats that received 2 mg/kg ABA daily for 7 days followed by 200 mg/kg CUR administered 2 h later (n = 9). The sixth group included rats that received 2 mg/kg ABA daily for 7 days followed by 20 mg/kg RES administered 2 h later (n = 9). One rat from each of these groups died during the experimental period.

The chemicals were dissolved in olive oil and administered via gavage. All rats were anesthetized with ketamine/xylazine and euthanized by cervical dislocation followed immediately by exsanguination 16 h after the last administration of ABA, CUR, or RES. On day 8, liver tissues were collected. At the end of the experiment, a portion of the liver tissue was set aside for histopathological evaluation, and the remaining tissue was used for biochemical analyses. The liver tissue intended for biochemical analyses was thoroughly washed with saline, blotted dry on filter paper, cut into small pieces, and weighed. A 10% (w/v) liver tissue homogenate was prepared with cold phosphate-buffered saline using an ultrasonic homogenizer (Bandelin SONOPLUS, Bandelin, Berlin, Germany). The homogenate was centrifuged at 10,000 rpm for 20 min at + 4 °C. The resulting liver tissue supernatants were transferred into Eppendorf tubes and stored at − 80 °C until use on the experimental day.

#### Biochemical analysis

The resulting supernatants were used for biochemical analyses. Levels of reduced glutathione (GSH) and lipid peroxidation (LPO), activities of catalase (CAT), superoxide dismutase (SOD), glutathione peroxidase (GPx), glutathione reductase (GR), and glutathione-S-transferase (GST), total oxidant status (TOS), and total antioxidant capacity (TAC) were determined. The oxidative stress index (OSI) was calculated. Total protein content was measured and used to normalize all biochemical parameters.

#### Determination of reduced glutathione (GSH) levels

GSH levels in liver tissue were determined using the method described by Beutler^24^. Proteins in the homogenates were precipitated with metaphosphoric acid, and GSH reacted with 5,5′-dithiobis-2-nitrobenzoic acid to form a colored complex. The absorbance of the resulting solution was measured spectrophotometrically at 405 nm. Results were expressed as nmol GSH/mg protein.

#### Determination of lipid peroxidation (LPO) levels

LPO levels in liver tissue were determined according to the method of Ledwozyw et al.^25^. The reaction between malondialdehyde (MDA) and thiobarbituric acid produced a pink chromogen, which was measured spectrophotometrically at 532 nm. Results were expressed as nmol MDA/g protein. For consistency, LPO results were normalized to protein content.

#### Determination of catalase (CAT) activity

CAT activity was determined by monitoring the decomposition of hydrogen peroxide (H₂O₂) to water and the corresponding decrease in absorbance at 240 nm^26^. Enzyme activity was expressed as U/mg protein.

#### Determination of superoxide dismutase (SOD) activity

SOD activity was determined based on its ability to inhibit the photooxidation of riboflavin-sensitive o-dianisidine. The assay mixture contained phosphate buffer (pH 7.8), o-dianisidine dihydrochloride, and riboflavin. After the addition of riboflavin, absorbance values were recorded at 460 nm under a fluorescent lamp before and 8 min after exposure^27^. Results were expressed as U/mg protein.

#### Determination of glutathione peroxidase (GPx) activity

GPx activity in liver tissue was determined using the method described by Wendel^28^. GPx catalyzes the oxidation of reduced glutathione (GSH) to oxidized glutathione (GSSG) in the presence of H₂O₂. The decrease in absorbance was measured spectrophotometrically at 366 nm. Results were expressed as U/mg protein.

#### Determination of glutathione reductase (GR) activity

GR activity was determined by measuring the rate of nicotinamide adenine dinucleotide phosphate (NADPH) oxidation during the reduction of GSSG at 340 nm^28^. Enzyme activity was expressed as U/g protein.

#### Determination of glutathione-S-transferase (GST) activity

GST activity was measured spectrophotometrically by monitoring the formation of the conjugate between GSH and 1-chloro-2,4-dinitrobenzene (CDNB) at 340 nm^29^. Results were expressed as U/mg protein.

#### Determination of total oxidant status (TOS)

Total oxidant status was measured using the method described by Erel^30^. The principle of the assay is based on the oxidation of ferrous ions $\left(\rm{Fe^{2+}}\right)$ to ferric ions $\left(\rm{Fe^{3+}}\right)$ by oxidants present in the sample under acidic conditions. The ferric ions then form a colored complex with xylenol orange, with a maximum absorbance at 580 nm. TOS levels were expressed as nmol H₂O₂ equivalents/g protein.

#### Determination of total antioxidant capacity (TAC)

Total antioxidant capacity was determined according to the method of Benzie and Strain^31^. In an acidic medium, antioxidants reduce ferric $\left(\rm{Fe^{3+}}\right)$-tripyridyltriazine (TPTZ) complex to ferrous $\left(\rm{Fe^{2+}}\right)$-TPTZ, resulting in the formation of a blue complex measured at 593 nm. TAC results were expressed as µmol/g protein.

#### Calculation of oxidative stress index (OSI)

The oxidative stress index (OSI) was calculated using the formula:

$$\mathrm{O}\mathrm{S}\mathrm{I}=\mathrm{T}\mathrm{O}\mathrm{S}\,\mathrm{\%}\,(\mathrm{T}\mathrm{A}\mathrm{C}\times 1000)$$

#### Determination of total protein content

Total protein content in liver tissue homogenates was determined according to the method of Lowry et al.^32^. This method is based on the formation of a blue-violet complex resulting from the reaction between proteins and copper ions under alkaline conditions, followed by reduction of the Folin–Ciocalteu reagent. Absorbance was measured spectrophotometrically at 500 nm using bovine serum albumin as the standard. Results are expressed as mg protein/g tissue and were used for normalization of biochemical parameters.

#### Histopathological evaluation

##### Tissue processing for light microscopy

Liver samples were collected from euthanized rats, cut into small pieces, and fixed in 10% neutral buffered formalin (HT5011228-4 L; Sigma–Aldrich, St. Louis, MO, USA) at room temperature for 24 h. After fixation, tissues were rinsed in distilled water, dehydrated through a graded ethanol series (70%, 90%, 96%, and 100%), cleared in xylene (399–5000; ChemSolute, Hilden, Germany), and infiltrated with paraffin in a 60 °C oven for 3–4 h before embedding. Paraffin blocks were sectioned at 5 µm thickness using a microtome (HM 340E; Thermo Scientific, Waltham, MA, USA). The sections were mounted on glass slides for routine histological staining and immunohistochemical analyses^33^.

##### Hematoxylin–eosin (H&E) staining

Sections were deparaffinized in xylene and rehydrated through a descending ethanol series, followed by staining using a commercial hematoxylin and eosin (H&E) kit (HAE-1-IFU; ScyTek, Logan, UT, USA). Hematoxylin was applied for 5 min at room temperature, after which slides were rinsed twice in distilled water, immersed in bluing solution for 15 s, and subsequently washed in 100% ethanol. Eosin Y was applied for 2 min 15 s, followed by three washes in 100% ethanol, clearing in xylene, and mounting with Bio-Mount medium (05-BMHM508; Bio-Optica, Milan, Italy). Histological images were acquired at 200 × magnification using a Nikon Eclipse light microscope (Nikon Instruments, Tokyo, Japan). Ten non-overlapping fields per section were evaluated for congestion, sinusoidal dilatation, inflammatory cell infiltration, and cytoplasmic vacuolization, each graded on a scale from 0 to 3 (none to severe). Histopathological scoring was applied to each animal group individually, and to facilitate clearer understanding of the scoring system, the evaluation criteria were organized and presented in a tabular format (Table 1). Group-level histopathological injury scores are presented in Fig. 5. The sum of these parameters was calculated as the total histological injury score^34,35^.

**Table 1:** Histopathological scoring parameters.

Score	Congestion	Cytoplasmic vacuolization	Sinusoidal dilatation	Inflammatory cell ınfiltration
0	None	None	None	None
1	Mild	Mild	Mild	Mild
2	Moderate	Moderate	Moderate	Moderate
3	Severe	Severe	Severe	Severe

##### TNF-α immunohistochemistry

Following deparaffinization in xylene and rehydration through a descending ethanol series, antigen retrieval was performed in 1 × citrate buffer (15-M103; Bio-Optica, Milan, Italy; diluted 1:10 in PBS) using a microwave oven (800 W for 3 min, followed by 180 W for 10 min). Slides were allowed to cool for 20 min at room temperature and washed three times for 5 min each in PBS (LRB05-100; Cistron, Istanbul, Turkey). Endogenous peroxidase activity was blocked using the peroxidase blocker provided in the HRP/AEC kit (AB93705; Abcam, Cambridge, UK) for 10 min, followed by protein blocking. Sections were then incubated overnight at 4 °C with an anti–TNF-α primary antibody (1:100; E-AB-33121; Elabscience, Houston, TX, USA). After washing in PBS, slides were incubated with a biotinylated goat anti-polyvalent secondary antibody for 10 min, washed again in PBS, and treated with streptavidin–peroxidase for 10 min. The AEC chromogen was developed for 8 min. Counterstaining was performed with Lillie’s modified Mayer’s hematoxylin for 2 min and bluing for 15 s, followed by rinsing with PBS and aqueous mounting (BMA-100; Biognost, Zagreb, Croatia). Four non-overlapping fields per sample were captured at 200 × magnification using the Nikon Eclipse light microscope (Nikon Instruments, Tokyo, Japan), and TNF-α immunoreactivity intensity was quantified using ImageJ software (National Institutes of Health, Bethesda, MD, USA)^36–38^.

### Statistical analysis

Statistical analyses were performed using GraphPad Prism 10.3.0 (GraphPad Software, San Diego, CA, USA). Biochemical data were analyzed using one-way analysis of variance (ANOVA) followed by Tukey’s multiple comparison test (p < 0.05). For histopathological assessment, four parameters were evaluated: congestion, cytoplasmic vacuolization, sinusoidal dilatation, and inflammatory cell infiltration. Ten randomly selected fields per liver section were imaged using a light microscope, scored individually, and the mean of the ten scores was taken as the histopathological score for each animal. Group differences were assessed using one-way ANOVA followed by Šidák’s multiple comparison test. For immunohistochemical analysis, four micrographs were captured from each liver section, and staining intensity was quantified using ImageJ (National Institutes of Health, Bethesda, MD, USA). Immunohistochemical data were also analyzed using one-way ANOVA with Tukey’s multiple comparison test. All results are presented as mean ± standard deviation (SD).

## Results

### Biochemical evaluations

**Fig. 1:**
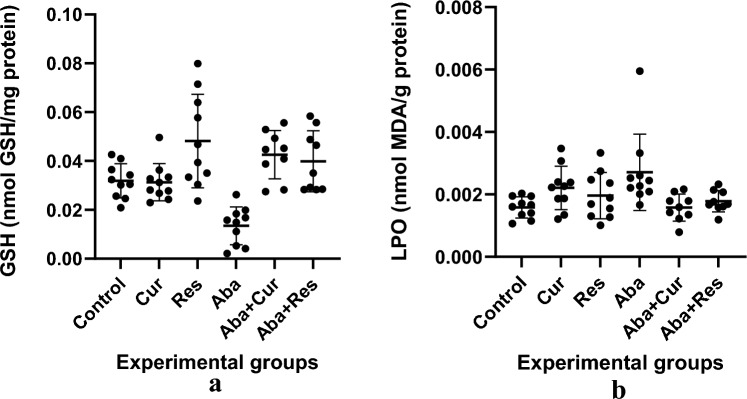
(**a**) Liver GSH Individual data points are shown. Horizontal lines indicate group means, and error bars represent standard deviation (SD). Sample sizes were n = 10 for Control, CUR, RES, and ABA groups, and n = 9 for the ABA + CUR and ABA + RES groups. (**b**). Liver LPO Individual data points are shown. Horizontal lines indicate group means, and error bars represent standard deviation (SD). An outlier was identified in the ABA group; statistical analyses were performed both including and excluding this value, and significance was maintained in both cases (p < 0.001 and p < 0.01, respectively). Sample sizes were n = 10 for Control, CUR, RES, and ABA groups, and n = 9 for the ABA + CUR and ABA + RES groups.

Compared with the control group, GSH levels significantly decreased in the ABA group (p < 0.001) and significantly increased in the RES group (p < 0.01), while no significant change was observed in the CUR group. Both the ABA + CUR and ABA + RES groups showed significant increases in GSH levels relative to the ABA group (p < 0.001) (Fig. 1a). LPO levels were significantly elevated in the ABA group compared with the control (p < 0.001), whereas no significant change was detected in the CUR or RES groups. Compared with the ABA group, both ABA + CUR and ABA + RES groups exhibited significant reductions in LPO levels (p < 0.01) (Fig. 1b). Visual inspection of the data revealed a potential outlier within the ABA group; therefore, statistical analyses were repeated after exclusion of this value. Following removal of the suspected outlier, LPO levels in the ABA group remained significantly higher than those in the control group, although the level of statistical significance was reduced (p < 0.01).

**Fig. 2:**
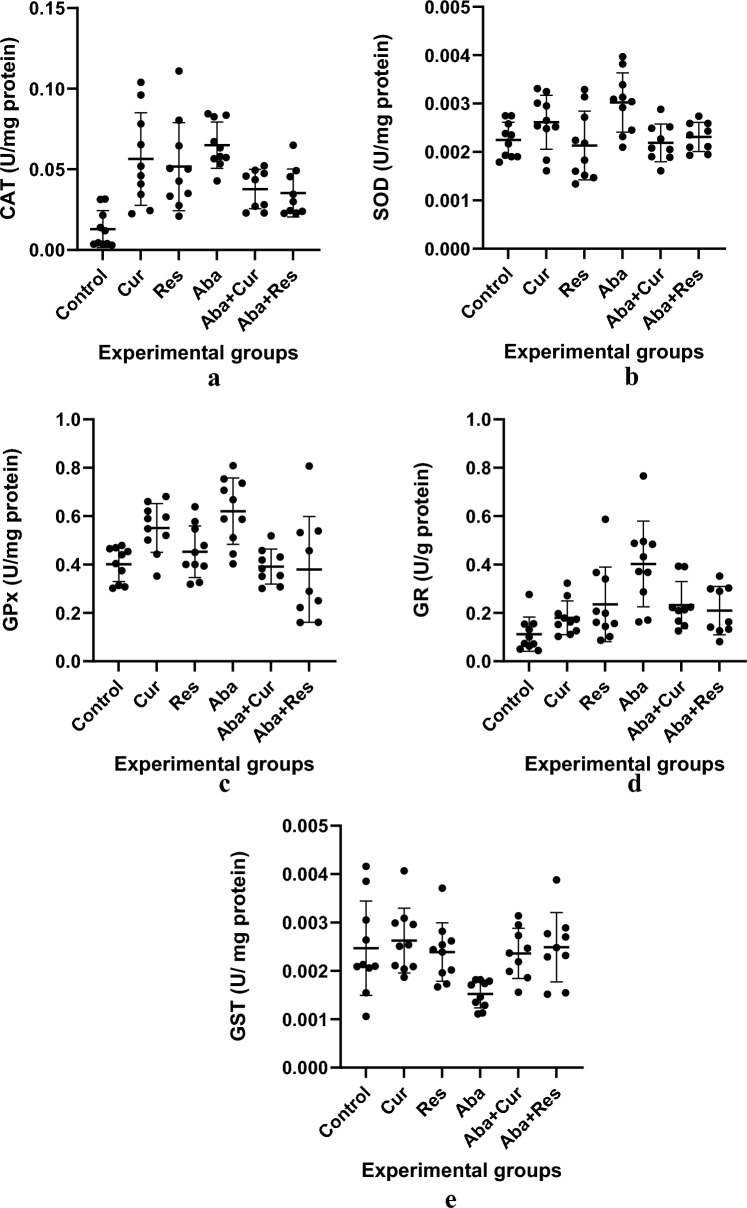
(**a**) Liver CAT Individual data points are shown. Horizontal lines indicate group means, and error bars represent standard deviation (SD). Sample sizes were n = 10 for Control, CUR, RES, and ABA groups, and n = 9 for the ABA + CUR and ABA + RES groups. (**b**) Liver SOD Individual data points are shown. Horizontal lines indicate group means, and error bars represent standard deviation (SD). Sample sizes were n = 10 for Control, CUR, RES, and ABA groups, and n = 9 for the ABA + CUR and ABA + RES groups. (**c**) Liver GPx Individual data points are shown. Horizontal lines indicate group means, and error bars represent standard deviation (SD). Sample sizes were n = 10 for Control, CUR, RES, and ABA groups, and n = 9 for the ABA + CUR and ABA + RES groups. (**d**) Liver GR Individual data points are shown. Horizontal lines indicate group means, and error bars represent standard deviation (SD). Sample sizes were n = 10 for Control, CUR, RES, and ABA groups, and n = 9 for the ABA + CUR and ABA + RES groups. (**e**) Individual data points are shown. Horizontal lines indicate group means, and error bars represent standard deviation (SD). Sample sizes were n = 10 for Control, CUR, RES, and ABA groups, and n = 9 for the ABA + CUR and ABA + RES groups.

Compared with the control group, the CUR group showed significant increases in CAT (p < 0.001) and GPx (p < 0.05) activities, with no significant changes in SOD, GR, or GST activities (Fig. 2a–e). The RES group similarly exhibited significant increases in CAT (p < 0.001) and GR (p < 0.05) activities, while SOD, GPx, and GST activities remained unchanged (Fig. 2a–e). In the ABA group, CAT (p < 0.001), SOD (p < 0.01), GPx (p < 0.001), and GR (p < 0.001) activities were all significantly elevated relative to the control, while GST activity was significantly decreased (p < 0.01) (Fig. 2a–e). Compared with the ABA group, both ABA + CUR and ABA + RES treatments resulted in significant decreases in CAT (p < 0.01), SOD (p < 0.001; p < 0.01), GPx (p < 0.001), and GR (p < 0.01; p < 0.001) activities, while significantly increasing GST activity (p < 0.01) (Fig. 2a–e).

**Fig. 3:**
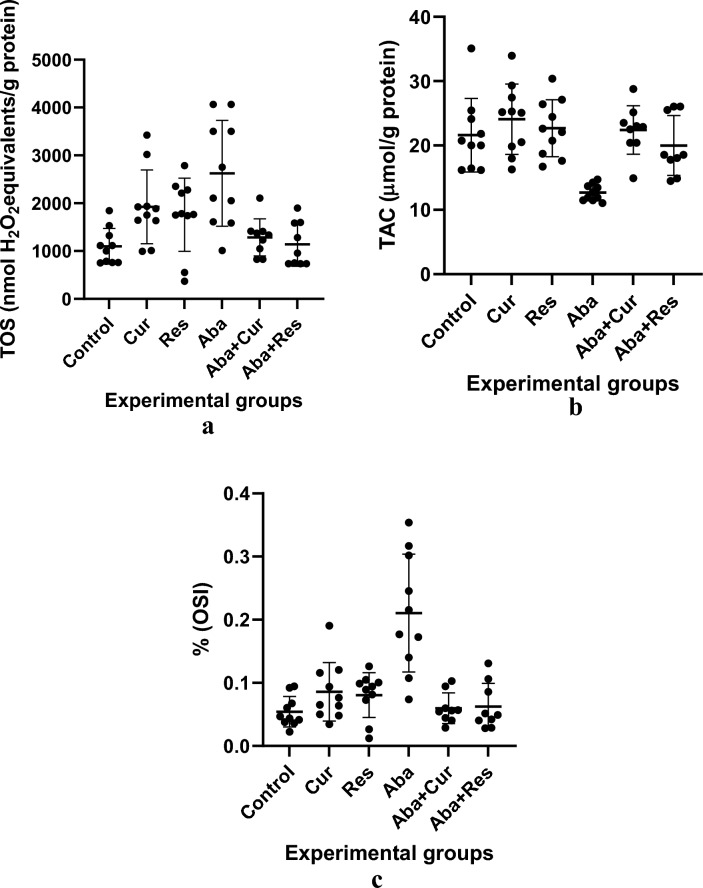
(**a**) Liver TOS Individual data points are shown. Horizontal lines indicate group means, and error bars represent standard deviation (SD). Sample sizes were n = 10 for Control, CUR, RES, and ABA groups, and n = 9 for the ABA + CUR and ABA + RES groups. (**b**) Liver TAC Individual data points are shown. Horizontal lines indicate group means, and error bars represent standard deviation (SD). Sample sizes were n = 10 for Control, CUR, RES, and ABA groups, and n = 9 for the ABA + CUR and ABA + RES groups. (**c**): Individual data points are shown. Horizontal lines indicate group means, and error bars represent standard deviation (SD). Sample sizes were n = 10 for Control, CUR, RES, and ABA groups, and n = 9 for the ABA + CUR and ABA + RES groups.

Relative to the control group, TOS and OSI values were significantly elevated in the ABA group (p < 0.001 for both), while TAC was significantly reduced (p < 0.001). Both ABA + CUR and ABA + RES groups showed significant decreases in TOS (p < 0.001) and OSI (p < 0.001) values, alongside significant increases in TAC (p < 0.0001; p < 0.001), compared with the ABA group (Fig. 3a–c).

### Histopathological evaluation

**Fig. 4:**
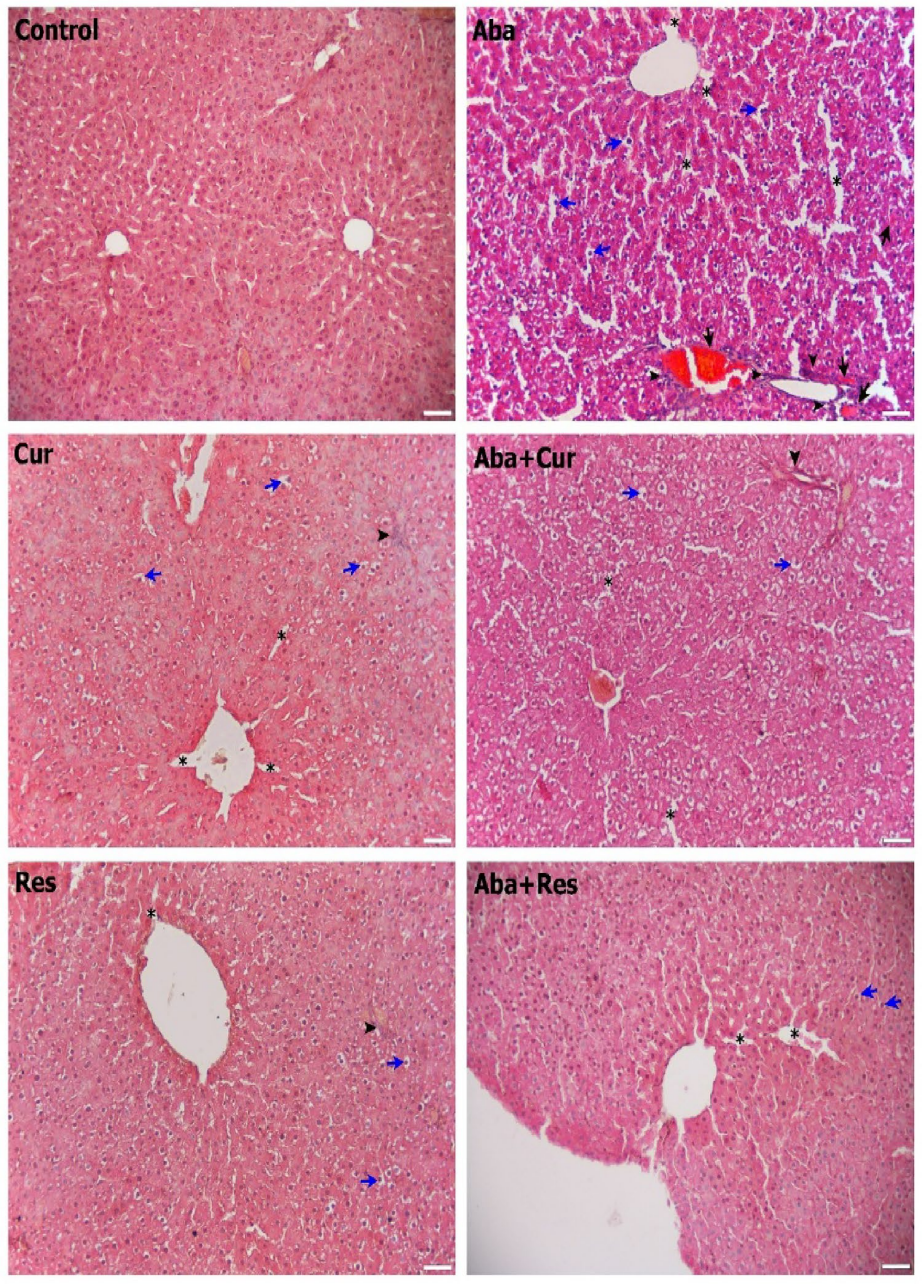
H&E-stained liver sections by group. Congestion is indicated by black arrows (→), sinusoidal dilatation by asterisks (*), vacuolization by blue arrows (** →**), and inflammatory cell infiltration by arrowheads (▸). 200 × magnification. Scale bar: 100 μm.

H&E staining revealed distinct histopathological alterations across the experimental groups (Fig. 4). The control group displayed normal hepatic architecture, while the ABA group exhibited prominent congestion, sinusoidal dilatation, inflammatory cell infiltration, and cytoplasmic vacuolization. The CUR and RES groups retained partial structural integrity with milder lesions. Treatment with CUR or RES following ABA exposure markedly attenuated hepatic injury compared with the ABA group alone, demonstrating the therapeutic efficacy of both compounds following ABA-induced hepatic injury.

Histopathological injury scores were significantly higher in the ABA group than in the control group (p < 0.001), confirming successful induction of hepatic damage. The CUR group showed a significantly elevated injury score relative to the control (p = 0.009), suggesting that CUR alone may exert mild hepatic stress under the experimental conditions employed. In contrast, the RES group did not differ significantly from the control (p > 0.05), indicating that RES alone does not elicit hepatotoxic effects at the administered dose.

**Fig. 5:**
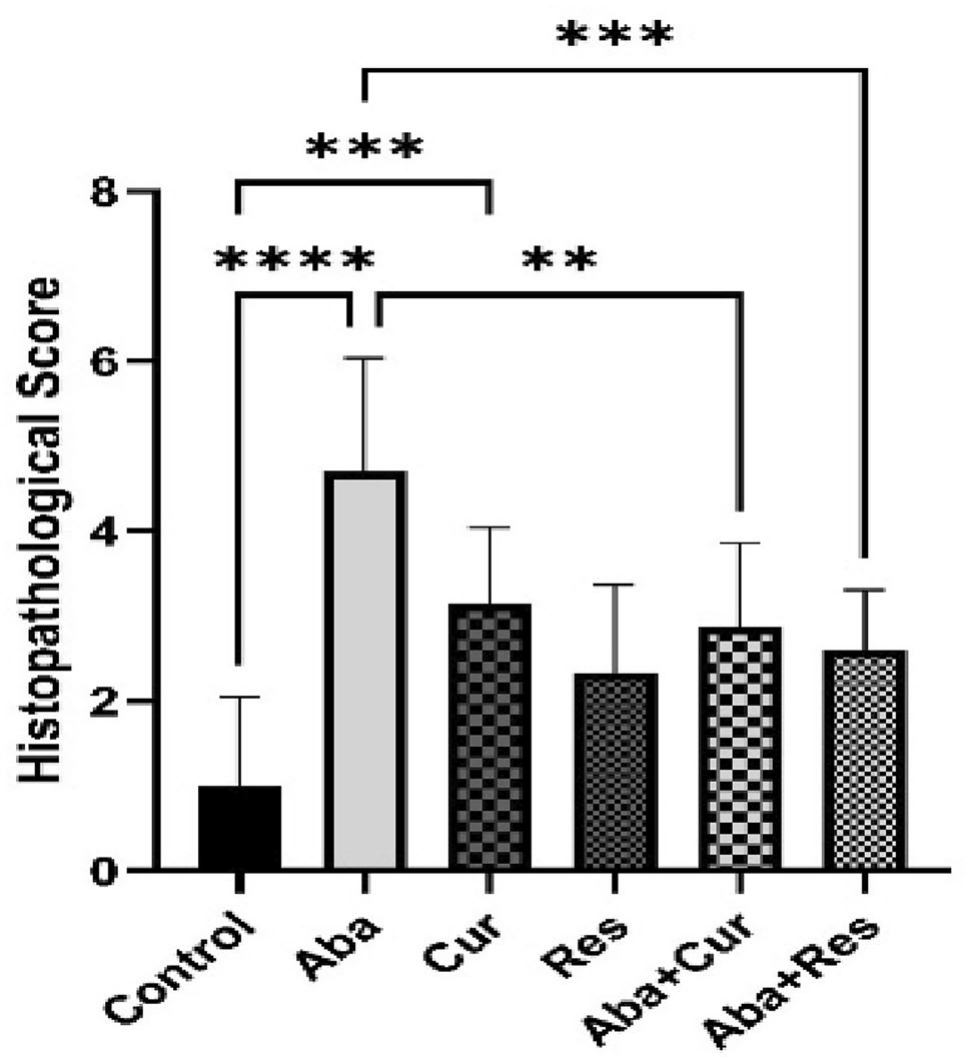
Comparison of total histopathological injury scores among the groups: Control, Abamectin (ABA), Curcumin (CUR), Resveratrol (RES), Abamectin + Curcumin (ABA + CUR), and Abamectin + Resveratrol (ABA + RES). ****p < 0.0001, ***p < 0.001, **p < 0.01. Sample sizes were n = 10 for Control, CUR, RES, and ABA groups, and n = 9 for the ABA + CUR and ABA + RES groups.

Following ABA exposure, both CUR (ABA + CUR) and RES (ABA + RES) treatment significantly reduced total histopathological injury scores compared with the ABA group (p < 0.01 and p < 0.001, respectively). Direct comparison between the ABA + CUR and ABA + RES groups revealed no significant difference in injury scores (p > 0.05), indicating that both compounds confer comparable hepatoprotective efficacy (Fig. 5).

### TNF-α Immunohistochemistry

**Fig. 6:**
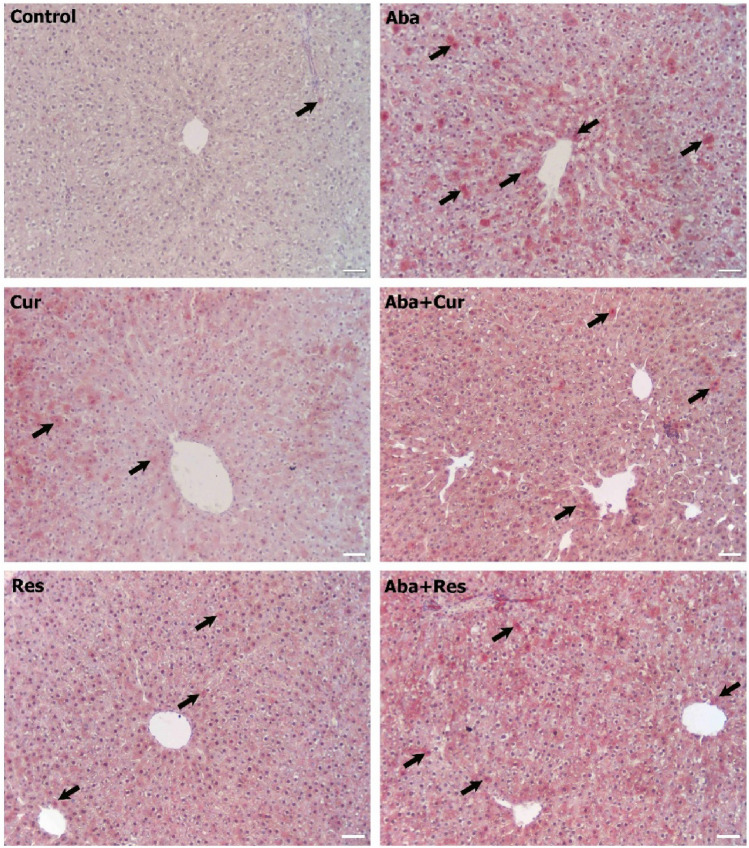
Liver sections subjected to TNF-α immunohistochemistry across experimental groups. Positive cells are indicated by black arrows (→). 200 × magnification. Scale bar: 100 μm.

Immunohistochemical analysis of TNF-α expression revealed distinct differences among the experimental groups in hepatic tissue (Fig. 6). The control group exhibited minimal cytoplasmic immunoreactivity, consistent with basal expression levels. In contrast, the ABA group showed intense and widespread TNF-α positivity, indicating marked inflammatory activation. The CUR and RES groups demonstrated low-to-moderate staining intensity. When co-administered with ABA, both CUR and RES markedly attenuated TNF-α expression compared with the ABA group, suggesting that antioxidant supplementation partially counteracts ABA-induced pro-inflammatory signaling.

**Fig. 7:**
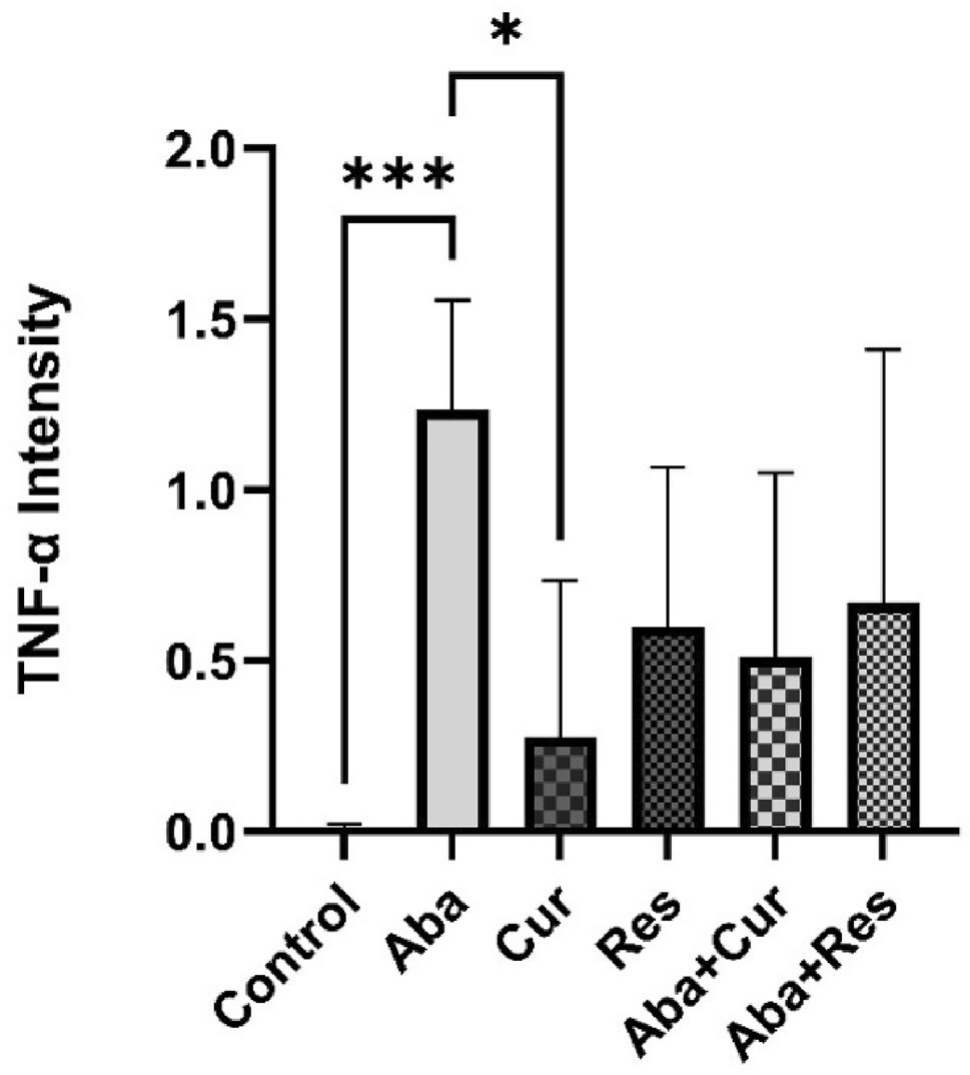
Comparison of intensity values based on TNF-α protein expression among the groups: Control, Abamectin (ABA), Curcumin (CUR), Resveratrol (RES), Abamectin + Curcumin (ABA + CUR), and Abamectin + Resveratrol (ABA + RES). ***p < 0.001, *p < 0.05. Sample sizes were n = 10 for Control, CUR, RES, and ABA groups, and n = 9 for the ABA + CUR and ABA + RES groups.

Quantitative analysis revealed that TNF-α expression was significantly higher in the ABA group than in the control group (p < 0.001), confirming a robust hepatic inflammatory response following ABA exposure. TNF-α immunoreactivity in the ABA group was also significantly greater than in the CUR group (p < 0.01), reflecting the more pronounced pro-inflammatory effect of ABA in the absence of antioxidant intervention. No significant difference in TNF-α expression was detected between the ABA and RES groups (p > 0.05). Although both co-treatment groups (ABA + CUR and ABA + RES) exhibited lower mean TNF-α levels compared with the ABA group, these reductions did not reach statistical significance (p > 0.05). Comparisons between the control group and either the CUR or RES groups likewise revealed no significant differences (p > 0.05). Taken together, these findings indicate that ABA markedly upregulates hepatic TNF-α expression, while the antioxidants tested showed only a non-significant trend toward attenuation under the present experimental conditions (Fig. 7).

### Correlation analysis

To assess relationships among histopathological parameters, a correlation analysis was performed. Only two positive correlations reached statistical significance: congestion vs. inflammatory cell infiltration (r = 0.89; 95% CI: 0.29–0.99; p = 0.017) and congestion vs. vacuolization (r = 0.81; 95% CI: 0.006–0.98; p = 0.049). These findings suggest that circulatory disturbances not only drive vascular changes but also promote parenchymal inflammatory and degenerative responses. The strong association of congestion with both inflammatory cell infiltration and cytoplasmic vacuolization points to a coordinated, system-level pathological process.

**Fig. 8:**
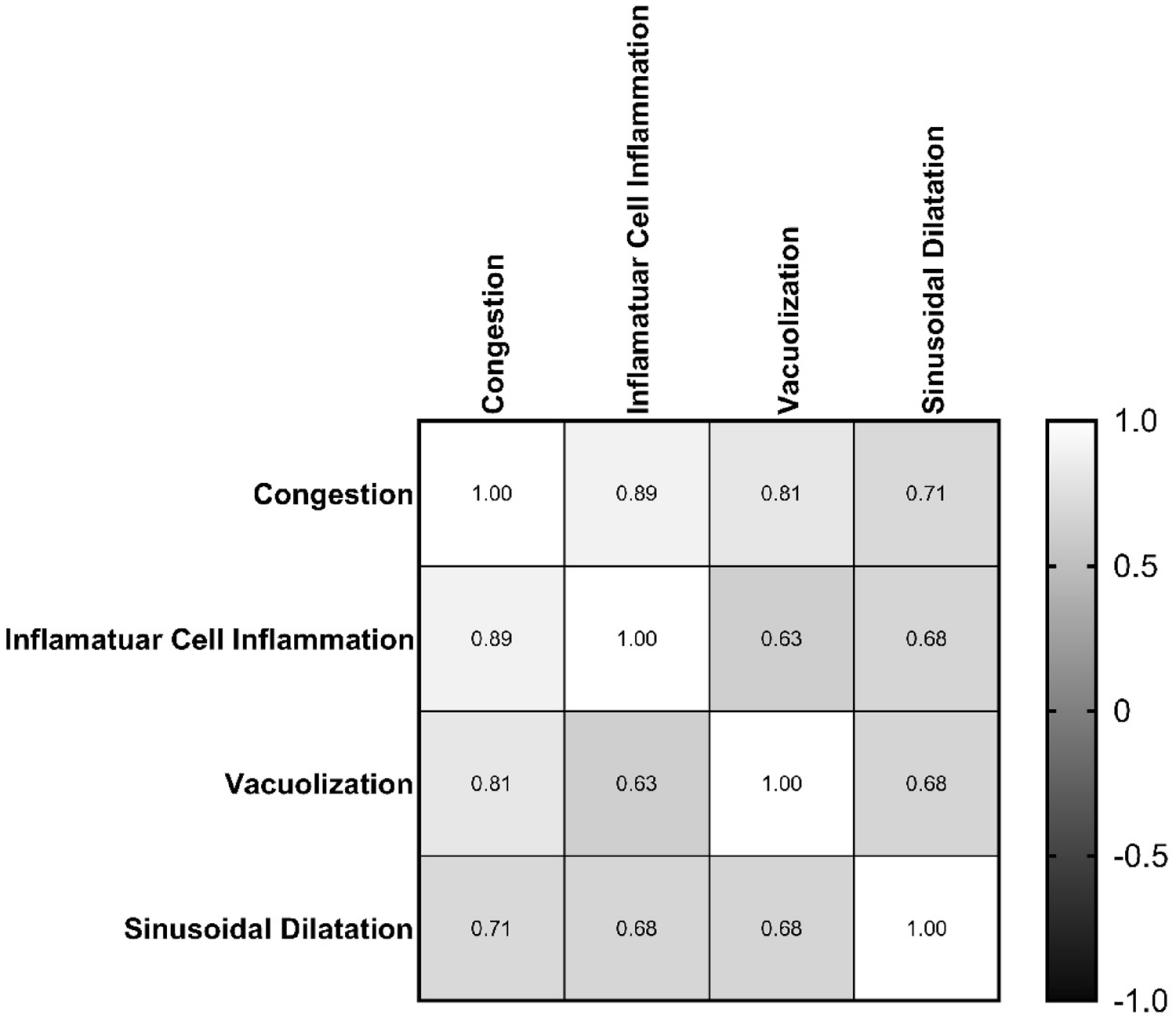
Correlation matrix of histopathological parameters. Pearson correlation coefficients (r) are shown among congestion, inflammatory cell infiltration, vacuolization, and sinusoidal dilatation. Color intensity is inversely proportional to correlation strength (lighter shades indicate stronger correlations).

By contrast, the correlations between congestion and sinusoidal dilatation (r = 0.71; 95% CI: − 0.24 to 0.96; p = 0.115), inflammatory cell infiltration and sinusoidal dilatation (r = 0.68; 95% CI: − 0.29 to 0.96; p = 0.137), and inflammatory cell infiltration and vacuolization (r = 0.63; 95% CI: − 0.37 to 0.95; p = 0.180) did not reach statistical significance, as their confidence intervals included zero. These results imply that although certain lesions tend to co-occur, inter-individual variability, limited sample size, and the dynamic progression of hepatic injury may obscure some expected associations (Fig. 8).

Overall, the strong correlations involving congestion underscore the central role of vascular disturbance in the pathogenesis of ABA-induced hepatic injury.

## Discussion

ABA is used as a pesticide in agriculture and for the treatment of helminth infections in humans and animals. ABA belongs to the avermectin family and is a 16-membered macrocyclic lactone with a disaccharide at the 13th carbon. In terms of its pesticide properties, abamectin consists of a minimum of 80% avermectin B1a and a maximum of 20% avermectin B1b^39^. Exposure to ivermectin and other macrocyclic lactones at nontherapeutic doses may cause toxic effects; however, these effects occur only after the ingestion of large quantities of these compounds. The mechanisms remain unclear, but at high doses, macrocyclic lactones can cross the blood–brain barrier and induce potentially toxic GABA-mimetic effects. The highest ABA levels have been detected in the liver and adipose tissue due to its lipophilic nature, whereas the lowest levels have been detected in the brain. Consequently, ABA toxification may impair hepatocyte function^6^. Oxidative stress is an important contributor to the cytotoxicity caused by avermectins^5^, and the use of antioxidants to mitigate this toxicity therefore represents a rational therapeutic approach.

Antioxidants play essential roles in scavenging ROS and preventing disease. Elevated ROS production can disrupt the GSH balance. GSH is a key cytoprotective biomolecule that acts against cytotoxicity through glutathione-S-transferase (GST)-mediated conjugation with electrophilic compounds. It also facilitates the rapid detoxification of hydrogen peroxide and lipid hydroperoxides via enzymatic (GPx) pathways^40^. Increased ROS levels trigger lipid peroxidation, and consequently MDA — the end product of polyunsaturated fatty acid peroxidation — serves as a reliable indicator of oxidative stress^41^.

In a study examining ABA-induced gastric toxicity, GSH levels decreased while MDA levels increased compared with controls^42^. Similarly, a study assessing oxidative stress parameters in rat liver reported decreased GSH alongside significantly elevated MDA in ABA-treated animals^43^. Another investigation of ABA-induced hepatotoxicity and nephrotoxicity likewise documented reduced GSH and elevated MDA relative to controls^23^. Consistent with these findings, our study demonstrated a significant decrease in GSH and a significant increase in LPO levels in the ABA group compared with the control group (p < 0.001) (Fig. 1a, b)**.** In the present analysis, visual inspection of the LPO dataset identified a potential outlier within the ABA group. Reanalysis following exclusion of this value resulted in a modest attenuation of the statistical significance (from p < 0.001 to p < 0.01), while preserving overall significance. This finding indicates that although a single extreme value contributed to the magnitude of the group effect, the elevation in LPO associated with ABA exposure was not solely driven by that observation (Fig. 1b).

The antioxidant efficacy of CUR and RES observed in our model is consistent with prior experimental literature. CUR has been reported to reduce MDA in models of cadmium and arsenic-induced liver toxicity^21^ and multiple studies confirm that CUR decreases MDA while augmenting GSH levels^44^^,^^45^. RES has similarly demonstrated redox-modulating properties,in an NMDA-induced retinal damage model, trans-resveratrol increased GSH and reduced MDA^46^, and Szkudelska et al. reported that RES contributes to reductions in MDA under diabetic conditions^47^. In line with these findings, both CUR and RES in the present study reversed the ABA-associated redox shift, producing significant increases in GSH and significant decreases in LPO in the co-treatment groups (p < 0.001**)** (Fig. 1a–b).

Beyond non-enzymatic defenses, first-line antioxidant enzymes including CAT, SOD, GPx, and GR play essential roles in cellular protection against reactive oxygen species^48^. GST additionally functions as a detoxifying enzyme by catalyzing glutathione conjugation with toxic intermediates, thereby limiting oxidative damage and contributing to intracellular signaling regulation^49^. In the present study, antioxidant enzyme responses exhibited enzyme-specific patterns rather than uniform changes. CAT activity showed significant increases in the CUR and RES groups compared with the control group and was also markedly elevated in the ABA group (Fig. 2a), indicating activation of antioxidant defenses under oxidative challenge. In contrast, SOD activity exhibited a statistically non-significant decrease in the RES group, while no significant change was observed in the CUR group (Fig. 2b). GPx activity was significantly increased in the CUR group, whereas only a non-significant increase was detected in the RES group (Fig. 2c). GR activity showed a significant increase in the RES group, while the corresponding increase in the CUR group did not reach statistical significance (Fig. 2d). GST activity displayed non-significant increases in both the CUR and RES groups relative to controls but was significantly decreased in the ABA group (Fig. 2e), reflecting impaired detoxification capacity under excessive oxidative burden. Collectively, these findings indicate that antioxidant enzyme responses to ABA exposure and polyphenol treatments are regulated in an enzyme- and compound-dependent manner rather than following a generalized unidirectional pattern.

Published findings regarding enzyme responses to ABA are, however, heterogeneous. Several studies report decreases in CAT and SOD activity in ABA-exposed rats^50^, as well as reduced CAT, SOD, and GPx in ABA-associated gastric toxicity^42^. Abdel-Daim and Abdellatief similarly reported decreases in CAT, SOD, and GPx in ABA-treated animals^23^. In contrast, Radi et al. observed increased CAT activity in rat liver under ABA exposure, which aligns more closely with our findings^43^. Many studies have reported higher CAT, SOD, GPx, and GR activities following CUR administration, consistent with our results^21,44,51^, while others have demonstrated that RES increases CAT and SOD activities^46^.

In our dataset, CAT activity was significantly elevated in the CUR, RES, and ABA groups compared with controls (Fig. 2a). SOD activity showed a non-significant decrease in the RES group, while no significant change was observed in the CUR group (Fig. 2b). GPx increased significantly in the CUR group while GR increased non-significantly; conversely, in the RES group, GPx increased non-significantly while GR increased significantly (Fig. 2c, d). GST activity increased non-significantly in both CUR and RES groups relative to controls but decreased significantly in the ABA group (Fig. 2e). Mechanistically, ABA may contribute to ROS redistribution through destabilization of lysosomal membranes and facilitate hydroxyl radical formation in hepatic tissue via diffusion of H₂O₂. Accordingly, despite the observed depletion of GSH, the concomitant rise in GSH-dependent enzymes (GPx and GR) may be interpreted as a compensatory response to increased free-radical generation. Both CUR and RES reversed these ABA-associated enzymatic derangements.

To further characterize ABA-induced oxidative stress, we quantified TOS, TAC, and OSI alongside enzyme activities. These parameters provide complementary measures of oxidative burden and antioxidant capacity, while OSI — calculated as the TOS/TAC ratio — offers an integrated index of systemic redox balance. Under oxidative stress, TAC typically declines while TOS increases, making OSI a sensitive readout of net redox disequilibrium^30^. In our study, TOS was significantly elevated in the ABA group compared with controls, while TAC was significantly reduced and OSI was significantly increased (Fig. 3a–c). Importantly, both ABA + CUR and ABA + RES significantly reduced TOS and OSI while significantly increasing TAC relative to the ABA group (Fig. 3a–c). Collectively, these results confirm that CUR and RES attenuate ABA-induced oxidative injury, and the discrepancies observed across studies likely reflect variation in dosing, treatment duration, and administration protocols.

Notably, when administered alone, both curcumin (CUR) and resveratrol (RES) resulted in a significant increase in total oxidant status (TOS) compared with the control group (Fig. 3a), accompanied by a non-significant increase in the oxidative stress index (OSI) (Fig. 3c). This apparently paradoxical response is consistent with accumulating evidence demonstrating that polyphenolic compounds can exert pro-oxidant or hormetic redox effects under specific conditions, particularly at higher doses or in the absence of an exogenous oxidative challenge^52^. Such dose- and context-dependent behavior has been attributed to auto-oxidation of phenolic moieties, formation of quinone intermediates, and transient increases in reactive oxygen species, mechanisms that have been described for both curcumin and resveratrol^53^. Importantly, the lack of a significant OSI elevation suggests that the observed rise in oxidant burden was at least partially counterbalanced by endogenous antioxidant capacity. Collectively, these findings highlight the biphasic redox nature of CUR and RES and underscore the importance of dose- and context-aware interpretation when evaluating their oxidative effects, providing a plausible explanation for their divergent impact when administered alone versus as co-treatments under overt oxidative stress conditions.

Our histopathological findings support a characteristic ABA-induced hepatic injury pattern dominated by microcirculatory stress — most notably sinusoidal congestion and dilatation — together with inflammatory cell infiltration and cytoplasmic vacuolization. The strong positive correlations between congestion and both inflammation and vacuolization suggest that vascular derangement may act as a proximate driver of parenchymal injury rather than a purely secondary phenomenon (Fig. 8). This interpretation aligns with established concepts in hepatic pathology, where sinusoidal congestion and dilatation often reflect impaired venous outflow or microvascular dysfunction and may accompany systemic inflammatory states^54^. Together, the data emphasize a hemodynamic–inflammatory axis as a central mechanism in ABA-mediated liver damage.

At the mechanistic level, ABA hepatotoxicity has been linked to oxidative stress and mitochondrial dysfunction, including enhanced CYP2E1 activity, lipid peroxidation, activation of caspase-3 and p38 MAPK pathways, and compromised mitochondrial bioenergetics^43^. These molecular perturbations align with the structural injury observed in our model and are concordant with the concomitant increase in TNF-α expression. Across experimental systems, avermectin-related toxicity converges on excessive ROS production and disturbed calcium homeostasis, providing a unifying framework for the vascular, inflammatory, and degenerative lesions observed here^7^.

Both CUR and RES significantly mitigated the composite histopathological injury induced by ABA (Fig. 4–5). This is consistent with extensive preclinical evidence that these polyphenols restore redox homeostasis and suppress NF-κB–mediated inflammatory signaling, in part through Nrf2 pathway activation. The hepatoprotective role of CUR has been comprehensively reviewed in the context of chronic liver diseases, further supporting its therapeutic relevance in hepatic injury models^55^. Nrf2 functions as a cellular defense orchestrator, upregulating antioxidant enzymes such as SOD, CAT, and GPx under conditions of oxidative stress, whereas NF-κB serves as a master regulator of pro-inflammatory genes including TNF-α^56^. These two pathways often counter-regulate one another: robust Nrf2 activity enhances antioxidant capacity and can constrain NF-κB-driven cytokine production, while unchecked NF-κB activation generates excess ROS and perpetuates inflammation, particularly when Nrf2 is concurrently suppressed^56–58^. For RES specifically, the SIRT1–NF-κB axis represents a particularly well-supported mechanism, with multiple studies demonstrating SIRT1-dependent repression of pro-inflammatory transcription coupled with improvements in hepatic structure and function^59^.

In our ABA model, this interplay appears evident. ABA exposure likely tips the balance by suppressing Nrf2 and hyperactivating NF-κB, as reflected in elevated hepatic TNF-α expression and widespread structural lesions in the ABA group (Fig. 6–7). By reactivating Nrf2 and attenuating NF-κB signaling, CUR and RES interrupt this cycle, thereby preserving hepatic architecture even when cytokine normalization remains incomplete. This dynamic explains why structural rescue can outpace full cytokine normalization and accounts for the comparable histological protection observed in the ABA + CUR and ABA + RES groups.

TNF-α immunolabeling revealed a more nuanced pattern. Although both CUR and RES produced a downward trend in TNF-α expression following ABA exposure, these reductions did not reach statistical significance (Fig. 6–7). Several methodological and biological factors may have contributed to this outcome: TNF-α expression is inherently dynamic and time-dependent; single-time-point sampling may overlook transient post-treatment declines; and statistical power may be sufficient for coarse histopathological endpoints but insufficient for quantitative intensity measurements. Additionally, semi-quantitative chromogenic IHC methods such as AEC-based detection are sensitive to staining variability and analytical parameters. The implementation of standardized digital pathology workflows — including QuPath-based color deconvolution with automated batch processing — could improve sensitivity and reproducibility for cytokine-related readouts in future studies^60^^,^^61^.

An additional finding warranting consideration is the mild histopathological worsening observed in the CUR-only group relative to controls. This pattern is compatible with the biphasic pharmacology of CUR. At conventional concentrations, CUR acts predominantly as an antioxidant and anti-inflammatory agent through Nrf2 activation and NF-κB suppression. However, under certain conditions — including high doses, specific light exposure, or distinct microenvironmental contexts — it can exert pro-oxidant effects or behave as a topoisomerase II poison, leading to DNA damage and apoptosis^56^. Singlet-oxygen–mediated photochemistry has also been proposed as a contributor to CUR’s pro-oxidant behavior^62^. Baseline tissue redox state, metal-ion availability, and formulation-specific properties may therefore plausibly explain the mild adverse structural signal observed in the CUR-only group.

A second clinically relevant consideration is the emerging evidence linking turmeric and CUR supplementation to rare, idiosyncratic drug-induced liver injury (DILI). Phytovigilance reports and case-series analyses describe acute hepatocellular or cholestatic hepatitis associated with high-bioavailability preparations, with improvement typically following discontinuation^63,64^. While the absolute number of reports remains limited and causality is still debated, curated reference resources such as LiverTox now list turmeric and CUR as suspected DILI-associated agents^63^. A recurrent factor in these reports is piperine co-formulation: pharmacokinetic studies have demonstrated that co-administration of piperine substantially increases CUR bioavailability^65^. Because piperine inhibits CYP3A4 and P-glycoprotein, drug–supplement interactions and elevated effective exposures become biologically plausible^21,44,68^^,^^69^ reinforcing that dose, formulation, and experimental context are key determinants of both safety and efficacy. These considerations do not diminish CUR’s therapeutic potential,rather, they underscore the importance of rigorous exposure characterization in experimental designs.

Finally, the congestion–dilatation phenotype observed in our model supports an integrated injury framework in which hemodynamic stress and inflammatory signaling — particularly through TNF-α and NF-κB–linked pathways — act synergistically to amplify parenchymal damage. Conversely, polyphenolic interventions can preserve hepatic architecture even when cytokine modulation remains incomplete^70^. Future studies would benefit from harmonized cytokine sampling schedules, explicit reporting of CUR formulation including piperine co-administration status, dosing within well-characterized exposure ranges, and standardized digital pathology workflows such as QuPath-based pipelines to better align structural and molecular endpoints^60^.

In summary, our data support a congestion-centered, redox-inflammatory model of ABA-induced hepatotoxicity in which both CUR and RES confer meaningful structural hepatoprotection. The mild adverse signal in the CUR-only group further highlights that hepatic outcomes are shaped by dose, bioavailability enhancers, and biological context — consistent with CUR’s biphasic antioxidant–pro-oxidant behavior and the emerging literature on rare idiosyncratic DILI^63^.

## Conclusion

In this rat model, ABA induced a reproducible hepatic injury pattern characterized by microvascular disturbance with secondary parenchymal involvement. Sinusoidal congestion and dilatation occurred alongside inflammatory cell infiltration and cytoplasmic vacuolization, and the degree of congestion closely correlated with the severity of inflammatory and degenerative changes. Collectively, this profile implicates impaired hepatic microcirculation as a principal driver of injury rather than a secondary consequence of parenchymal damage. Biochemical analyses further confirmed that ABA exerts significant hepatotoxic effects under the present experimental conditions.

When administered after ABA, both CUR and RES attenuated structural injury and preserved hepatic architecture to a comparable extent. TNF-α expression increased following ABA exposure and exhibited only a partial reduction in the ABA + CUR and ABA + RES groups, indicating that structural recovery can precede full cytokine normalization. This dissociation reflects the capacity of antioxidant and anti-inflammatory pathways — including redox buffering and modulation of the NF-κB, Nrf2, and SIRT1 axes — to stabilize the hepatic microenvironment even when individual inflammatory markers remain elevated. Consistent with these structural findings, both CUR and RES also reversed the ABA-induced alterations in biochemical parameters, including GSH, LPO, antioxidant enzyme activities (CAT, SOD, GPx, GR, and GST), as well as TOS, TAC, and OSI.

Two observations refine the safety and translational interpretation of our findings. First, CUR alone produced slightly higher injury scores than the control group, supporting the concept that CUR’s effects are dose-, context-, and formulation-dependent. This finding suggests that the dose used in the present experimental model represents a non-clinical exposure scenario and may not directly reflect typical clinical dosing conditions. Second, RES alone appeared neutral under the present conditions. Taken together, these results support CUR and RES as therapeutic adjuncts for the management of ABA-induced liver injury, while underscoring the need for judicious CUR use — particularly in high-bioavailability formulations. Clinically, these findings reinforce therapeutic strategies aimed at mitigating hepatic oxidative and inflammatory stress, provided that formulation and dosing are carefully optimized to balance efficacy and safety.

### Limitations

This study has certain limitations that should be acknowledged. The experimental design included only male Wistar albino rats to minimize hormonal variability. Estrogen has been reported to have antioxidant and hepatoprotective properties, which could potentially attenuate oxidative stress–related damage and obscure the assessment of abamectin-induced hepatotoxicity as well as the effects of curcumin and resveratrol. Although this approach improved experimental control, it limits the generalizability of the findings. Future studies including female animals are therefore warranted to better understand sex-related differences.

Another limitation of this study is that standard serum liver function biomarkers (ALT, AST, and ALP) were not evaluated. Although blood samples were collected during the experimental period, hemolysis was observed in a substantial proportion of the serum specimens, and hemolysis is known to adversely affect the reliable measurement of these enzymes. Therefore, serum samples were excluded from biochemical analyses to avoid misleading results. Future studies employing non-hemolyzed serum samples are warranted to provide a more comprehensive assessment of hepatocellular injury.

## Supplementary Information

Supplementary Information 1

Supplementary Information 2

## Data Availability

All data generated or analysed during this study are included in this published article.
