# Supplementary material for: Ameliorative effects of curcumin and resveratrol following abamectin-induced liver injury in rats
Source: Sci Rep. 2026 May 26;16:24104. doi: 10.1038/s41598-026-54518-9 (PMC13439200; doi:10.1038/s41598-026-54518-9)
Supplement: Supplementary file 1 [file 41598_2026_54518_MOESM1_ESM.pdf]

T.C.
İSTANBUL MEDİPOL ÜNİVERSİTESİ
Hayvan Deneyleri Yerel Etik Kurulu (Med-Hadyek) Kararı

Toplantı Tarihi	Karar No	İlgi	Proje Yürütücüsü
08/07/2024	38		Neziha HACIHASANOĞLU ÇAKMAK

"Abamektin ile oluşturulan karaciğer hasarına Kurkumin ve Resveratrol'ün Etkilerinin Araştırılması" başlıklı bilimsel araştırma etik kurulumuzda görüşülmüş olup, çalışmanın etik kurallara uygun olduğuna "**Oybirliği**" ile karar verilmiştir.

Etik Onay Geçerlilik Süresi: 5 yıl

GÖREVİ	ADI SOYADI	İMZA
Başkan	Dr. Öğr. Üyesi Muhammed İkbal ALP	
Üye	Prof. Dr. İlknur KESKİN	
Üye	Doç. Dr. Ayşe Arzu ŞAKUL	
Üye	Dr. Öğr. Üyesi Neşe AYŞİT	
Üye	Dr. Öğr. Üyesi Salih GENCER	
Üye	Dr. Öğr. Üyesi Serdar ALTUNAY	
Üye	Uzm.Vet. Hek. Ekrem Musa ÖZDEMİR	
Üye	Cem GÜNEŞ	
Üye	Burak Sefa DERİBAŞ	
