# Supplementary material for: Ameliorative effects of curcumin and resveratrol following abamectin-induced liver injury in rats
Source: Sci Rep. 2026 May 26;16:24104. doi: 10.1038/s41598-026-54518-9 (PMC13439200; doi:10.1038/s41598-026-54518-9)
Supplement: Supplementary file 2 [file 41598_2026_54518_MOESM2_ESM.pdf]

T.C.
İSTANBUL MEDİPOL ÜNİVERSİTESİ
Hayvan Deneyleri Yerel Etik Kurulu Başkanlığı

Sayı : E-38828770-302.14.06-4079 08/07/2024
Konu : Etik Kurul Kararı

Sayın Neziha HACIHASANOĞLU ÇAKMAK

Üniversitemiz Hayvan Deneyleri Yerel Etik Kuruluna yapmış olduğunuz "Abamektin ile Oluşturulan Karaciğer Hasarına Kurkumin ve Resveratrol'ün Etkilerinin Araştırılması" isimli başvurunuz incelenmiş olup, etik kurulu kararı ekte sunulmuştur.

Bilgilerinize sunarım.

Dr. Öğr. Üyesi Muhammed İkbal ALP
Hayvan Deneyleri Yerel Etik Kurulu
(MED-HADYEK) Başkanı

EK:
Etik Kurul Kararı

Bu belge, güvenli elektronik imza ile imzalanmıştır.
Evrağınızı https://turkiye.gov.tr/istanbul-medipol-universitesi-ebys linkinden F1697E8EX7 kodu ile dogrulayabilirsiniz.

Medipol Üniversitesi Kavacık Yerleşkesi (Ana Yerleşke Rektörlük)
Kavacık Mah. Ekinciler Cad. No: 19, Kavacık Kavşağı, 34810 Beykoz,İstanbul
T: 444 85 44 F: 0212 531 75 55
E-Posta: bilgi@medipol.edu.tr İnternet Adresi: www.medipol.edu.tr
Kep Adresi: medipoluniversitesi@hs03.kep.tr

Ayrıntılı Bilgi İçin: Fatma GÜZHAN
Tel: 5771 **E-Posta**: Fatma.guzhan@medipol.edu.tr
